# Routine health data describe adherence and persistence patterns for oral diabetes medication for a virtual cohort in the Khayelitsha sub-district of Cape Town, South Africa

**DOI:** 10.1371/journal.pgph.0002730

**Published:** 2023-12-21

**Authors:** Tsaone Tamuhla, Peter Raubenheimer, Joel A. Dave, Nicki Tiffin

**Affiliations:** 1 Division of Computational Biology, Integrative Biomedical Sciences Department, Faculty of Health Sciences, University of Cape Town, Cape Town, South Africa; 2 South African National Bioinformatics Institute, University of the Western Cape, Cape Town, South Africa; 3 Division of Endocrinology, Department of Medicine, Faculty of Health Sciences, University of Cape Town (UCT), Cape Town, South Africa; 4 Wellcome Centre for Infectious Disease Research in Africa, Institute of Infectious Diseases and Molecular Medicine, University of Cape Town, Cape Town, South Africa; PLOS: Public Library of Science, UNITED STATES

## Abstract

Type 2 diabetes mellitus (T2DM) is managed with combined lifestyle modifications and antidiabetic drugs, but people on treatment often fail to reach glycaemic control. Adherence is important for achieving optimal glycaemic control, and management of diabetes with drugs is a lifelong process, so understanding adherence through analysis of longitudinal medications data is important. Using retrospective routine health data and metformin dispensing records as a proxy for medication use, we describe longitudinal persistence and adherence to oral diabetes medication in a virtual cohort of 10541 people with diabetes (PLWD) in Khayelitsha subdistrict, Cape Town. Adherence was measured in 120-day sliding windows over two years and used to estimate metformin adherence trajectories. Multinomial logistic regression identified factors influencing these trajectories. Analysis of pharmacy dispensing records showed varying medication refill patterns: while some PLWD refilled prescriptions consistently, others had treatment gaps with periods of non-persistence and multiple treatment episodes–from one to five per individual across two years. There was a general trend of decreasing adherence over time across all sliding windows in the two-year period, with only 25% of the study population achieved medication adherence (> = 80% adherence) after two years. Four adherence trajectories; ‘low adherence gradual decline (A), ‘high adherence rapid decline’ (B), ‘low adherence gradual increase (C) and ‘adherent’ (D) were identified. Only trajectory D represented participants who were adherent at treatment start and remained adherent after two years. Taking HIV antiretroviral treatment before or concurrently with diabetes treatment and taking metformin in combination with sulphonylurea and/or insulin were associated with the long-term adherence (trajectory D). Routine data shows real life medication implementation patterns which might not be seen under controlled study conditions. This study illustrates the utility of these data in describing longitudinal adherence patterns at both an individual and population level.

## Introduction

Diabetes is one of the fastest growing global health threats with an estimated 1 in 10 people currently living with the disease [1]. Type 2 diabetes mellitus (T2DM) is the most prevalent form of diabetes accounting for more than 90% of all cases [2–5]. Chronic hyperglycaemia increases the risk of developing micro- and macro-vascular complications which are associated with increased morbidity and mortality in people living with diabetes (PLWD) [3–5]. To minimise the risk of developing complications, T2DM is managed using a combination of lifestyle modifications and antidiabetic medications with the aim of achieving glycaemic control—established as a glycated haemoglobin (HbA1c) of less than 7% [6]. Antidiabetic medications can effectively reduce hyperglycaemia and improve glycaemic control [6–10]. Studies have shown, however, that PLWD who are on treatment often fail to reach glycaemic targets [9, 11–18]. While there are several factors that can contribute to lack of glycaemic control including the natural progression of the disease, one of the key factors is non-adherence to diabetes treatment [11, 19, 20]: treatment non-adherence has been shown to negatively impact treatment efficacy and lead to increased morbidity and mortality in PLWD [12, 21, 22].

Sub-Saharan Africa (SSA) currently has the lowest prevalence of diabetes in the world, but, has the highest diabetes-related morbidity and mortality in people under 60 years [1]. Non-adherence to diabetes treatment has also been widely studied in SSA, but most of the data have been from cross sectional studies. These studies have reported varying trends of adherence ranging from as low as 25% to as high as 75% and identified several factors including sex, socio-economic status, age, high cost of medication, comorbidities, pill burden, medication availability, drug side-effects and being asymptomatic as having an influence on adherence to antidiabetic medication PLWD [23–28]. While these studies have provided valuable insight about adherence to diabetes treatment PLWD in SSA, they are limited in that they rely on patient self-reporting, a subjective measure that is prone to bias and only estimates adherence at a single time point. Management of diabetes is a lifelong process, and an individual’s medication adherence patterns can change over time, therefore, it is important to estimate adherence using longitudinal data [29, 30].

There is a paucity of data on longitudinal adherence patterns to diabetes medication PLWD in SSA. This is worrying in a region that is predicted to soon have an exponential increase in diabetes prevalence [1]. Given the significance of adherence in achieving optimal glycaemic control, estimating longitudinal adherence can better elucidate temporal adherence patterns which can be more informative and useful for improved patient care and targeted interventions [29, 30]. In the current study we explore methods to use longitudinal routine health data to describe adherence and persistence patterns for diabetes medication use. We then use these methods to better understand adherence and persistence patterns to metformin in a virtual cohort of people with T2DM who are starting their diabetes treatment in the Western Cape Province of South Africa.

## Methods

### Ethics statement

Ethics approval was granted by the University of Cape Town (HREC REF: 509/2019) and data access was approved by Western Cape Government Health (WCGH), South Africa. All data were de-identified and data perturbation was employed by the Provincial Health Data Centre (PHDC, WCGH) prior to release, so that the data used were anonymised and cannot be reidentified. Data transfer was effected through secure platforms using AES256 encryption and password protection, and analysis was undertaken on a secured, firewall-protected server. Re-use of this dataset requires approval from WCGH and contact details to apply for access are provided in the data availability statement. There was no patient and public involvement in this research due to the anonymised and perturbed nature of the data.

### Study population

The study population was selected from a pre-existing dataset of public health care seekers in the Khayelitsha sub-district in the Western Cape Province. The Khayelitsha subdistrict is a high-density urban area with generally poor socioeconomic conditions and a high burden of TB and HIV. The study population in the pre-existing dataset were selected from the Western Cape population as represented in the PHDC, a health information exchange containing routine health data for about 7 million healthcare clients, collated daily from multiple electronic health data sources in the Western Cape Province, South Africa [31]. Inclusion criteria in the pre-existing dataset were: (1) Having attended at least one Government Health Facility in the Khayelitsha sub-district in the Western Cape, South Africa, in the period 1 January 2016 to 31 December 2017, and (2) aged 18 or older by December 2017.

In the current study, inclusion criteria were: (1) a diagnosis of diabetes inferred from PHDC records using listed disease evidences of at least one glycated haemoglobin (HbA1c) value greater than or equal to 6.5% [32], fasting glucose results, and/or dispensed diabetes drugs, and (2) diabetes medication dispensed at health facilities linked to electronic routine pharmacy records. Exclusion criteria were: (1) no recorded dispensing of metformin in the study observation window, (2) diabetes ascertainment at less than 18 years of age and diabetes treatment using insulin only, used as a proxy for early onset Type 1 Diabetes, (3) diabetes ascertainment occurring during pregnancy, used as a proxy for gestational diabetes, (4) diabetes treatment start after 31^st^ March 2018 as there would be insufficient data for a two-year follow-up and (5) diabetes treatment start before 1^st^ January 2011 as there would be insufficient data in the PHDC records.

The study had a two-year window for observing medication dispensing patterns for PLWD in the Khayelitsha sub-district. Diabetes treatment start date was defined as day 0 in the observation window and all individuals were followed up for two-years from their diabetes treatment start date. The study analysed retrospective routine health data and used recorded medication dispensing episodes as a proxy for medication use. Since metformin is a first-line drug for treating type 2 diabetes [33–35], metformin dispensing was used as a proxy for diabetes treatment.

Retrospective PHDC data for 10541 individuals who were recorded as having started diabetes treatment on metformin were analysed together with population demographics assessed as of 31 December 2017, using descriptive statistics. A diagnosis is inferred by the PHDC using laboratory and pharmacy data and is not a clinical diagnosis made during a consultation, so is referred to as ‘ascertainment’ to make this distinction. The process of episode ascertainment is described in further detail in [31, 36]. Diabetes metrics include: ‘Diabetes ascertainment age’, ‘Diabetes treatment initiation age’ which was defined as the participant age at the recorded diabetes treatment start date, ‘Diabetes treatment initiation’ which was inferred from the PHDC data by calculating the time interval in years between diabetes ascertainment date and diabetes treatment start date, ‘Diabetes treatment formulation’ which was defined as the combination of the different classes of diabetes medication dispensed to an individual in the study observation window, ‘Hypertension’ which was defined as hypertension ascertainment before or during the study observation window identified by dispensing of anti-hypertensive medication, and ‘Tuberculosis’ which was defined as a Tuberculosis episode ascertained during the study observation window, and ‘HIV positive’ which was defined as HIV infection ascertainment before or during the study observation window.

In South Africa, HIV is managed through a parallel, vertically funded, well-resourced chronic disease programme with a large focus on adherence, which may impact adherence to other chronic disease including diabetes. Since the Khayelitsha sub-district has a high burden of HIV, to determine if there were differences in adherence based on HIV antiretroviral therapy (ART) use, we compared the sub-populations who were identified as using HIV ART vs those who were not. ‘HIV antiretroviral therapy’ use was defined as having started HIV ART before or during the study observation window.

Summary statistics were calculated for the study population. For continuous data, median and interquartile range were calculated and for grouped data, percentages were calculated.

### Diabetes treatment persistence and adherence

The R statistical software [37] package *AdhereR* [38] was used to calculate persistence and adherence to diabetes oral drugs in the study population. ‘Persistence’ was defined as a period of continuous medication dispensing with treatment gaps of less than 90 days [38, 39]. ‘Treatment gaps’ were defined as the time interval between dispensing events where no medication was dispensed, and in this study if a treatment gap was equal to or exceeded 90 days this was defined as ‘non-persistence’ or ‘treatment discontinuation’ [38]. If an individual was then dispensed medication following a period of ‘non-persistence’, this was treated as a new ‘treatment episode’ and the number of treatment episodes was determined by how often an individual discontinued and re-started treatment in the observation window [38].

‘Adherence’ was defined as how well the treatment regimen was implemented and it was calculated for each specified medication dispensing observation window [38, 39]. Since we were interested in how well the treatment regimen was implemented in the first two years following the first recorded treatment start episode, we calculated adherence to metformin in successive 4 month intervals or ‘sliding windows’ over the two year observation window [38]. Sliding window adherence was used to elucidate any temporal changes in adherence which would not be observed if one overall adherence measure was used [38]. Some patients were on more than one diabetes oral drug during the observation window, but adherence was calculated for only the drug metformin to avoid over-estimating adherence, given that *AdhereR* does not have the functionality to distinguish concurrent medication use in a treatment episode [38]. In this way, we used metformin as an index medication for measuring T2DM treatment adherence in PLWD.

### Adherence trajectories

Metformin adherence trajectories for the two-year observation window were estimated from the sliding window adherence estimates using the R [37] package *kml* [40, 41]. The *kml* package applies *k*-means clustering to longitudinal data and clusters it into groups with similar characteristics [40, 41]. The *k*-means clustering algorithm was implemented as previously described [41], and the number of runs to determine each cluster partition was set to 200. Following cluster assignment, summary statistics were calculated for the sub-populations in each cluster. For continuous data, median and interquartile range were calculated and for grouped data, proportions were calculated. Wilcoxon rank sum was used to calculate significance of difference in median values between clusters, and Fisher’s exact test to calculate significance of difference in proportions between clusters. Multinomial logistic regression was used to determine which factors influenced adherence trajectory in individuals in the study population, using the defined clusters as the dependent variable (outcome) and demographic and comorbidity profiles as independent variables (risk factors).

### Monitoring treatment outcome using HbA1c

HbA1c testing is the gold standard for monitoring glycaemic control in PLWD, particularly those on treatment [32, 42]. To determine the implementation of HbA1c testing in the study population, counts of patients with HbA1c measures were done at baseline and at six-month intervals for the duration of the two-year study observation window. Baseline HbA1c was defined as the latest HbA1c test result up to 3 months before treatment was initiated. Median HbA1c measures were also calculated for those with available data. Summary statistics were calculated for the sub-populations in each trajectory cluster. For continuous data, median and interquartile range were calculated and for grouped data, proportions were calculated. Wilcoxon rank sum was used to calculate significance of difference in median values between clusters and Fisher’s exact test to calculate significance of difference in proportions between clusters.

### Health care utilisation

The number of health facility encounters for each study participant was calculated for a three-year period starting from the six months prior to the diabetes treatment initiation until 6 months after the treatment observation window and used as a proxy for health care utilisation. During the two-year observation window, health facility encounters were counted in four month sliding windows. Counts of the total number of participants with health facility encounters were done and median encounters were also calculated.

## Results

### The study population

There were 16979 individuals with an inferred diabetes episode, of which 10541 met the described inclusion/exclusion criteria and were included in the study population. Most of the study population (53.7%) initiated diabetes treatment at diabetes ascertainment while 25.6% initiated treatment more than one year after diabetes ascertainment. Hypertension (61.8%), HIV (14.9%) and Tuberculosis (13.1%) were the most prevalent comorbidities with available data. In this study population, only 76.5% of people living with HIV (PLWHIV) had initiated HIV anti-retroviral treatment (S1 Table) before or during the two-year study observation window.

### Diabetes treatment

PLWD in this study population were treated with metformin, sulphonylurea (gliclazide, glimepiride or glibenclamide) and insulin. During the two-year observation window, 2803 (30.2%) participants were treated with metformin only, 4109 (44.3%) with metformin and sulphonylurea and 2372 (25.5%) with metformin, sulphonylurea and insulin (Table 1). Comparing HIV-negative and HIV-positive groups showed no difference in the proportion of people on the different diabetes treatment formulations between the two groups in this study population (S1 Table). However, there was a significant difference in diabetes treatment initiation with a higher proportion of the HIV-positive group initiating treatment within one year of diabetes ascertainment compared to the HIV-negative group (S1 Table).

**Table 1:** Characteristics of the study population stratified by longitudinal metformin adherence trajectory.

	Whole study population N = 10541	Adherent (D) N = 1544	Low adherence gradual decline (A) N = 4656	High adherence rapid decline (B) N = 2716	Low adherence gradual increase (C) N = 1625
Sex: Female	7053 (67.0%)	1014 (65.8%)	3136 (67.4%)	1830 (67.5%)	1073 (66.2%)
Diabetes Ascertainment Age (Years)	52.0 [44.0;59.0]	49.0 [42.0;57.0]	53.0 [45.0;61.0]	52.0 [45.0;59.0]	51.0 [44.0;58.0]
Diabetes Treatment Initiation Age (Years)	53.0 [45.0;60.0]	50.0 [43.0;57.0]	54.0 [45.0;62.0]	53.0 [46.0;60.0]	52.0 [44.0;59.0]
Diabetes Treatment Formulation:					
Metformin only	3525 (33.4%)	274 (17.7%)	2029 (43.6%)	756 (27.8%)	466 (28.7%)
Metformin & Sulphonylurea	4417 (41.9%)	770 (49.9%)	1682 (36.1%)	1188 (43.7%)	777 (47.8%)
Metformin, Sulphonylurea & Insulin	2599 (24.7%)	500 (32.4%)	945 (20.3%)	772 (28.4%)	382 (23.5%)
Diabetes Treatment Initiation:					
At diabetes ascertainment	5828 (55.3%)	863 (55.9%)	2693 (57.8%)	1352 (49.8%)	920 (56.6%)
Within 1 year of ascertainment	2156 (20.5%)	313 (20.3%)	926 (19.9%)	553 (20.4%)	364 (22.4%)
More than 1 year after ascertainment	2557 (24.3%)	368 (23.8%)	1037 (22.3%)	811 (29.9%)	341 (21.0%)
HIV Antiretroviral Therapy:	1202 (11.4%)	317 (20.5%)	472 (10.1%)	236 (8.7%)	177 (10.9%)
HIV Positive:	1572 (14.9%)	352 (22.8%)	651 (14.0%)	336 (12.4%)	233 (14.3%)
Hypertension:	6517 (61.8%)	933 (60.5%)	2749 (59.0%)	1728 (63.6%)	1107 (68.1%)
Tuberculosis:	1385 (13.1%)	236 (15.3%)	647 (13.9%)	325 (12.0%)	177 (10.9%)

### Diabetes treatment implementation

#### Persistence

**Fig 1:**
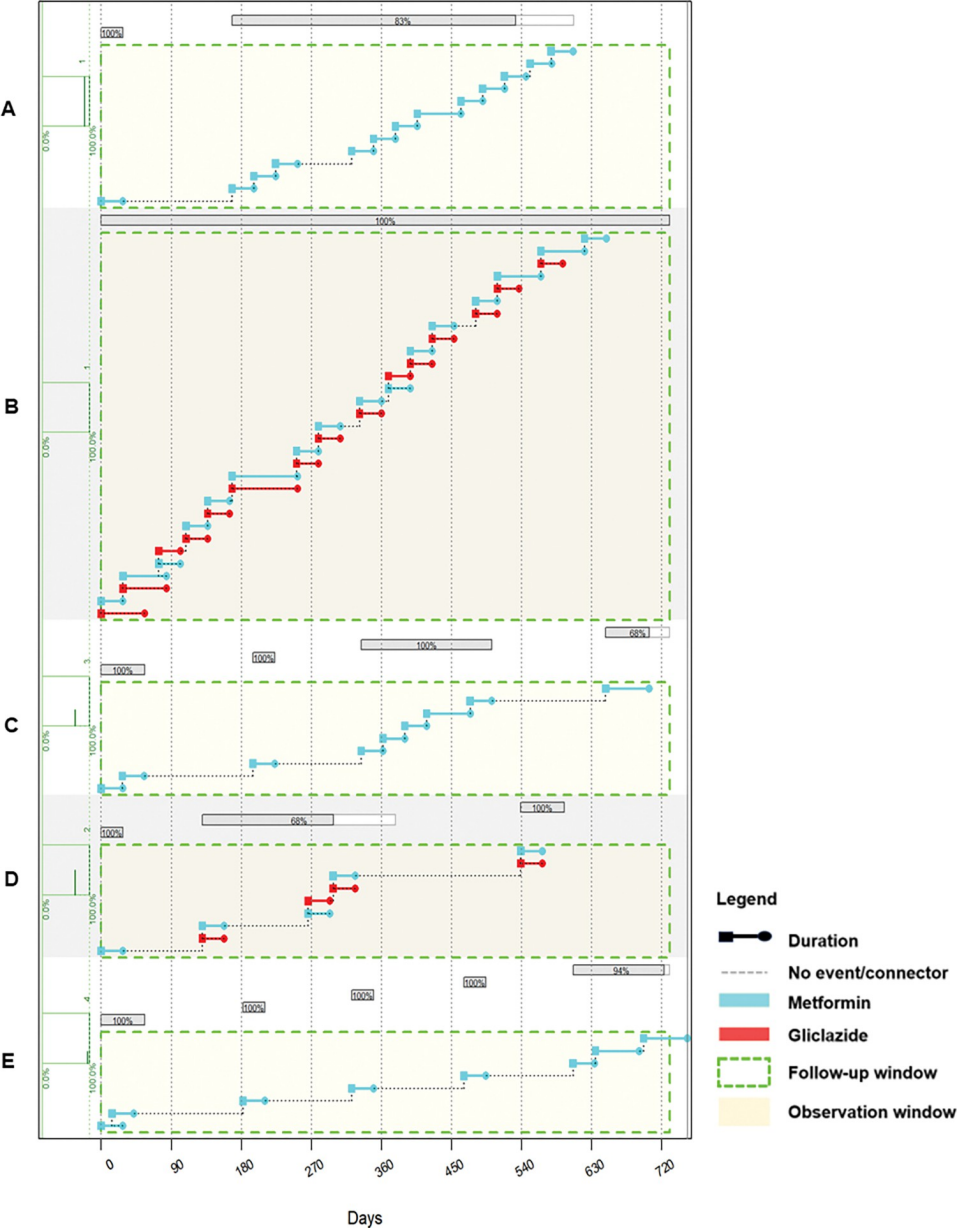
Diabetes oral medication refill patterns in five example patients in the study population. All oral diabetes medication (metformin and sulphonylurea) issued to patients during the observation window are shown. A: This shows an individual who is mostly adherent—the grey bar at the top of the plot indicates 83–100% adherence, with only metformin dispensed. Two interruptions in treatment are indicated by dotted lines between turquoise bars indicating treatment events. B: This shows an individual with 100% adherence on both gliclazide and metformin treatments. C: This shows an individual with long treatment interruptions in the first year of treatment followed by an improvement in the last part of the observation period. E: This individual has very poor adherence and is only receiving intermittent metformin treatment as shown by the multiple dotted lines between metformin dispensing.

An analysis of medication refill patterns in the study population in the two-year observation window showed that some individuals refilled their medication consistently while others had gaps in treatment. These gaps in treatment resulted in periods of non-persistence and multiple treatment episodes across the two years assayed which ranged in number from one to as high as five in some participants. Medication refill patterns of five study participants are illustrated in the plots in Fig 1 to illustrate the types of profiles seen in the study population: participant D had three treatment episodes, the first was at treatment initiation with a 28-day supply of metformin following which they had no recorded medication refill for 102 days which resulted in a period of non-persistence. Following treatment re-initiation, in the second treatment episode, the treatment formulation was changed to include a sulphonylurea (gliclazide). While this treatment episode was longer than the first, it also had medication refill gaps which resulted in the calculated adherence for the episode being 68% shown in the grey bar (Fig 1). The participant then had another period of non-persistence, following which they re-initiated treatment resulting in a third treatment episode. In contrast, participant B refilled their medication consistently and did not have any periods of non-persistence during the two-year observation window (Fig 1).

#### Adherence

**Fig 2:**
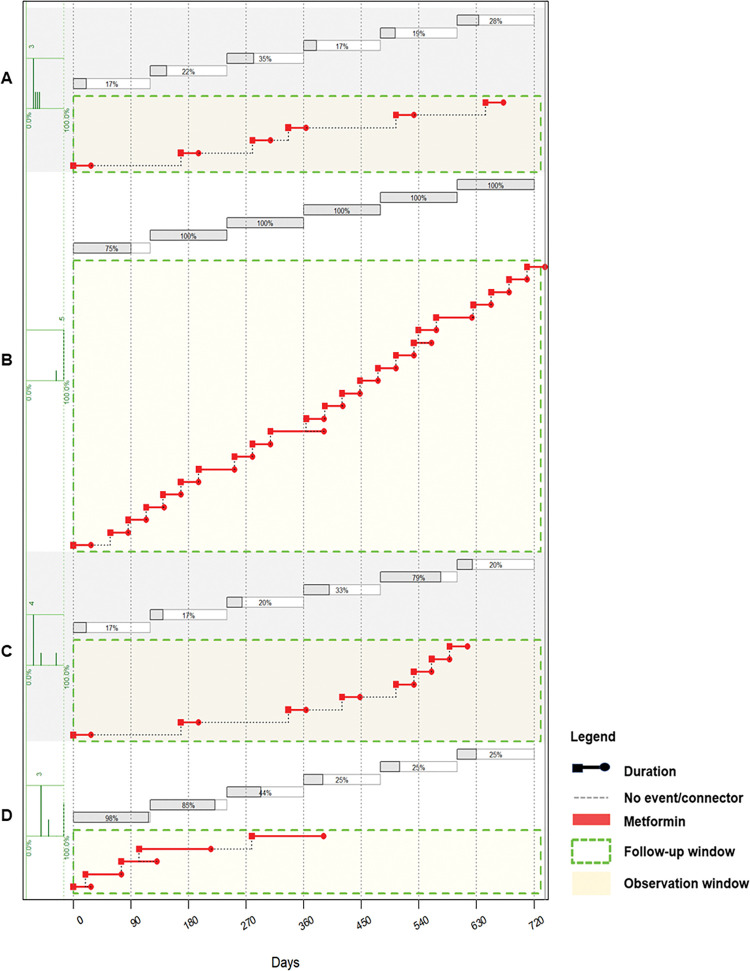
Longitudinal metformin adherence calculated in 120-day sliding windows in a two-year observation window. Adherence for each 120-day sliding window is shown in grey bars above the plots. The results of four patients from the study population are shown. A: This individual shows consistently poor adherence, with a maximum adherence of 35% for any sliding window. B: This individual is consistently adherent. C: This individual initially shows poor adherence, but their adherence improves from 17% to 79% in the later part of the two-year period. D: This individual begins their treatment with high adherence up to one year of treatment, but by the second year of treatment does not receive any further treatment.

Since we observed that the study participants had varying medication refill patterns, we calculated adherence to metformin in the two-year observation window in 120-day sliding windows. The results in Fig 2 show that while some individuals, like participant B, had consistent adherence measures in the different sliding windows, others had varying levels of adherence at different time points in the two-year observation window. The 120-day sliding window adherence measures show that participant A oscillated between 17–35% adherence, while participant D started at 98% adherence in the first 120 days and their adherence measures gradually decreased with each subsequent sliding window to as low at 25% after 360 days on diabetes treatment (Fig 2).

#### Longitudinal adherence patterns

**Fig 3:**
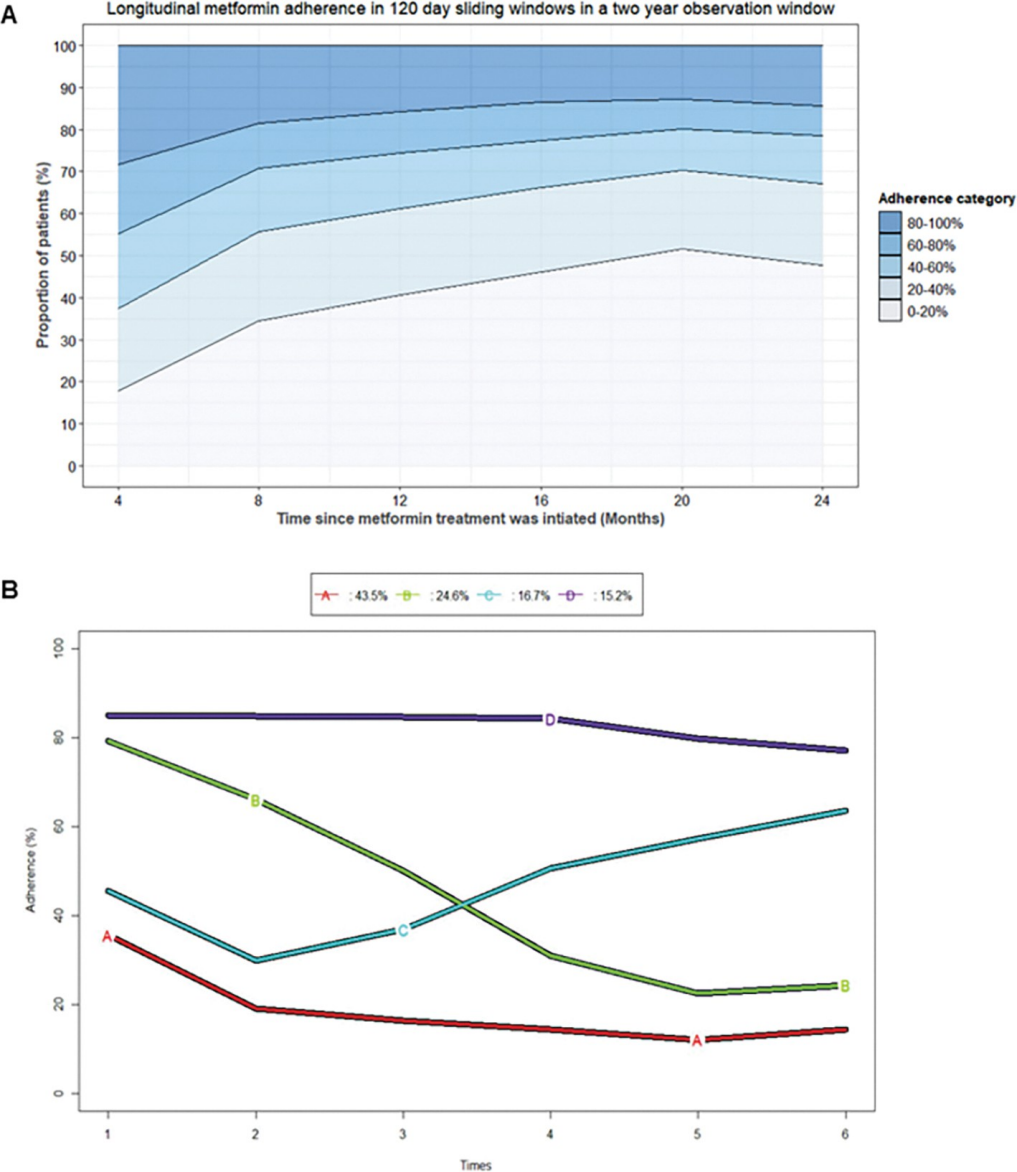
**(A).** A stacked area chart showing the proportion of patients in different adherence categories to visualise longitudinal trends in patient adherence. Each line represents the proportion of patients at a given time point and the colour below the line represents the corresponding adherence category. **(B).** Predicted two-year longitudinal metformin adherence trajectories for individuals in the study population. Four trajectories are shown: A (Low adherence gradual decline), B (High adherence rapid decline), C (Low adherence gradual increase) and D (Adherent). Adherence is shown on the y-axis and the four-month sliding windows are represented as times 1–6 on the x-axis. The percentage bar shows the proportion of patients in each adherence trajectory.

There is a general trend of decreasing adherence over time and across all sliding windows in the two-year observation window and by the end of the two-year period only 25% of the population achieved medication adherence (> = 80% adherence) (Fig 3A).

In addition, the proportion of adherent patients reduced with each subsequent sliding window and the lowest numbers (11.2%) were observed at 20 months post treatment initiation. Most of the non- adherent participants had adherence measures in the 0–20% adherence category across all sliding windows over the two-year observation period and proportion of patients with 0–20% adherence measures increased steadily over time from 27.5% at 4 months to 57.4% at 20 months post treatment initiation (Fig 3A). In the 20–24 month sliding window, we observed a slight decrease in the proportion of non-adherent participants, however, the proportion of participants who were adherent was approximately half (12.7%) of what it was 4 months after initiating treatment (25.0%) (Fig 3A).

#### Longitudinal metformin adherence trajectories

The longitudinal clustering of the 120-day sliding window adherence measures for each patient in the study population produced clusters with between 2 to 6 partitions, however, there was discordance among the five-criterion used to determine cluster partition choice (S1 Fig). The two-cluster partition which represents the typical binary outcome of adherent or non-adherent had the highest score, but in this study, we selected the four-cluster partition as the choice that best represented the study setting (Fig 3B) because we are interested in a more granular classification to understand the range/levels of adherence or non-adherence. In this approach, the adherent and non-adherent categories are further broken down to create four clusters consisting of one adherent cluster and 3 that represent non-adherence at different levels. The cluster partitioning of the 4 clusters converged on one distribution, whereas the 3, 5 and 6-cluster options had alternative compositions which made them less reliable.

In the four-cluster partition, the highest proportion of study participants (44.2%) were assigned to “trajectory A” which represented individuals who initiate treatment with low adherence measure that continue to decline gradually over time. “Trajectory B” had 25.8% membership and represents participants who were adherent in the first four months of treatment (Fig 3B), but their adherence declined rapidly thereafter and continued to decline over time. “Trajectory C” which had a membership of 15.4% was made up of participants who start treatment with low adherence, but over time have a gradual improvement in adherence. “Trajectory D” which has the lowest membership (14.6%) is the only trajectory with individuals that start treatment adherent and maintain their adherence long term, even though they start to decline slightly after month 16 (Fig 3B). The results in Fig 3B also show that for all trajectories except D, there is a sharp decline in adherence between the 1^st^ and 2^nd^ sliding window, following which trajectory C has an improvement while trajectories A and B continue to decline.

#### Characteristics of the adherence trajectory sub-populations

Comparing the four adherence trajectory groups (Table 1) showed that there were differences in age at diabetes treatment initiation between the groups, with the adherent group (D) having the youngest median age at treatment initiation of 49 years (IQR: 42,57). There were also differences in diabetes treatment formulation between the groups with the low adherence-gradual decline group (A) having the highest proportion of individuals (43.6%) on metformin only. There were also differences in diabetes treatment initiation with the high adherence-rapid decline group (B) having the highest proportion of individuals (29.9%) who initiated treatment more than a year after diabetes ascertainment. There was also a difference in the prevalence of hypertension between the groups with the low adherence-gradual increase group (C) having the highest proportion of individuals who were ascertained with hypertension (68.1%). In addition, trajectory D, the adherent group, had the highest proportion of participants who were HIV positive (22.8%) and on HIV antiretroviral therapy (ART) (20.5%).

#### Predictors for longitudinal diabetes treatment adherence trajectory

*Low adherence gradual decline (A) vs Adherent (D)*. Treatment age (OR: 1.02, 95% CI: 1.01–1.03), HIV ascertainment (OR: 1.70, 95% CI: 1.16–2.50) or TB ascertainment (OR: 1.28, 95% CI: 1.07–1.53) before and/or concurrently with diabetes treatment were associated with a higher likelihood of an individual having adherence trajectory A when compared to trajectory D. HIV antiretroviral treatment (OR: 0.26, 95% CI: 0.17–0.39) or hypertension ascertainment (OR: 0.77, 95% CI: 0.68–0.87) before and/or concurrently with diabetes treatment was associated with a lower likelihood of having adherence trajectory A when compared to trajectory D. When compared to individuals on metformin only, people who were on metformin & sulphonylurea (OR: 0.29, 95% CI: 0.25–0.34) or metformin, sulphonylurea & insulin (OR: 0.25, 95% CI: 0.21–0.30) were all associated with a lower likelihood of having adherence trajectory A when compared to trajectory D. Sex and time of treatment initiation in relation to ascertainment were not associated with patient adherence trajectory (S2 Fig).

*High adherence rapid decline (B) vs Adherent (D)*. Treatment age (OR: 1.01, 95% CI: 1.00–1.02) or HIV ascertainment before and/or concurrently with diabetes treatment (OR: 1.56, 95% CI: 1.05–2.31) were associated with a higher likelihood of a patient having adherence trajectory B when compared to trajectory D. When compared with initiating treatment at diabetes ascertainment, treatment initiation more than one year after diabetes ascertainment (OR: 1.43, 95% CI: 1.23–1.67) was associated with a higher likelihood of an individual having trajectory B when compared to trajectory D. Taking HIV antiretroviral treatment before or concurrently with diabetes treatment (OR: 0.26, 95% CI: 0.17–0.40) was associated with a lower likelihood of having trajectory B when compared to trajectory D. When compared to individuals on metformin only, people who were on metformin & sulphonylurea (OR: 0.54, 95% CI: 0.45–0.63) or metformin, sulphonylurea & insulin (OR: 0.52, 95% CI: 0.44–0.63) had a lower likelihood of having adherence trajectory B when compared to trajectory D. Sex, hypertension, tuberculosis and treatment initiation within one year of diabetes ascertainment were not associated with patient adherence trajectory (S2 Fig).

*Low adherence gradual increase (C) vs Adherent (D)*. Hypertension ascertainment (OR: 1.28, 95% CI: 1.10–1.50) before and/or concurrently with diabetes treatment was associated with a higher likelihood of a patient having adherence trajectory C when compared to trajectory D. Taking HIV antiretroviral treatment before or concurrently with diabetes treatment (OR: 0.35, 95% CI: 0.22–0.55) was associated with a lower likelihood of a having trajectory C when compared to trajectory D. When compared to individuals on metformin only, people who were on metformin & sulphonylurea (OR: 0.59, 95% CI: 0.49–0.71) or metformin, sulphonylurea & insulin (OR: 0.46, 95% CI: 0.38–0.57) had a lower likelihood of having adherence trajectory C when compared to trajectory D. Sex, tuberculosis, treatment initiation, treatment age and HIV were not associated with patient adherence trajectory (S2 Fig).

### Monitoring glycaemic control using HbA1c

8474 study participants had at least one HbA1c measure in the two-year observation period, however only 26.7% (2262) had a baseline HbA1c before initiating diabetes treatment (Table 2). The median HbA1c at baseline was above 9.0% for all adherence trajectories and the highest in trajectory D at 10.9% (IQR: 8.60, 13.0) and the lowest in trajectory A at 9.1% (IQR: 7.30, 11.3). While there was a general trend of decreasing median HbA1c across all adherence trajectories in the two-year observation, it was still above 8.0% across all trajectories. In addition, there was also a general trend of low HbA1c implementation with less than 35% of the study population having an available HbA1c measure in each of the sampled time periods during the two-year observation window (Table 2). When looking at HbA1c implementation in general, the proportion of study participants with at least one HbA1c in the first year following diabetes ascertainment was less than 90% across all adherence trajectories and there was a gradual decrease in the number of individuals with recorded HbA1c measures in each successive year. After 5 years less than 40% of the study population had a recorded HbA1c measure (S2 Table).

**Table 2:** HbA1c measures and proportions of study participants with available HbA1c measures in the period before treatment initiation (Baseline) and in six-month intervals during the two-year observation window.

	Whole study population *N = 8474*	Adherent (D) *N = 1442*	Low adherence gradual decline (A) *N = 3152*	High adherence rapid decline (B) *N = 2444*	Low adherence gradual increase (C) *N = 1436*	N^¥^
Baseline HbA1c (%), median [IQR]	9.7 [7.6;11.9]	10.9 [8.6;13.0]	9.1 [7.3;11.3]	9.9 [7.8;12.3]	9.6 [7.8;11.7]	2262
Participants with baseline HbA1c, N (%)	2262 (26.7%)	339 (23.5%)	945 (30.0%)	585 (23.9%)	393 (27.4%)	
Six-month HbA1c (%), median [IQR]	9.1 [7.3;11.5]	9.7 [7.7;11.7]	8.6 [7.0;11.1]	9.5 [7.4;11.7]	8.8 [7.2;11.5]	2328
Participants with six-month HbA1c, N (%)	2328 (27.5%)	440 (30.5%)	769 (24.4%)	765 (31.3%)	354 (24.7%)	
One-year HbA1c (%), median [IQR]	8.7 [7.1;11.0]	8.7 [7.2;10.9]	8.4 [7.0;10.8]	8.8 [7.1;11.5]	8.9 [7.1;10.9]	2694
Participants with one-year HbA1c, N (%)	2694 (31.8%)	539 (37.4%)	887 (28.1%)	844 (34.5%)	424 (29.5%)	
Eighteen-month HbA1c (%), median [IQR]	8.5 [7.1;10.9]	8.5 [7.1;10.7]	8.6 [7.0;11.0]	8.5 [7.1;10.7]	8.6 [7.1;10.9]	2157
Participants with eighteen-month HbA1c, N (%)	2157 (25.5%)	398 (27.6%)	636 (20.2%)	632 (25.9%)	491 (34.2%)	
Two-year HbA1c (%), median [IQR]	9.0 [7.2;11.3]	9.2 [7.3;11.4]	8.9 [7.2;11.2]	8.9 [7.2;11.3]	9.1 [7.3;11.3]	2597
Participants with two-year HbA1c, N (%)	2597 (30.6%)	490 (34.0%)	859 (27.3%)	757 (31.0%)	491 (34.2%)	

¥Number of participants with available data

Across all adherence trajectories general health care utilisation was highest in the first four months after treatment initiation (S3 Table). Trajectory A dropped to 67.2% after 8 months and maintained a health care utilisation of between 60–70% for the duration of the study observation window. Trajectories B, C and D all maintained a health utilization above 80% up to 16 months in the study observation period, but by the end of the two-year period, only Trajectory C still had a health care utilisation above 80% (S3 Table).

## Discussion

Pharmacy dispensing records from administrative health data have been widely used to estimate adherence to antidiabetic medication because they allow for the use of objective measures of adherence such as medication possession ratio (MPR) and proportion of days covered (PDC) [8, 12, 29, 43–45]. In the current study we similarly used pharmacy dispensing records as a proxy for medication use and used a modified continuous measure of medication acquisition (CMA) to estimate adherence to metformin. The modified CMA was used because it allowed for the estimation of longitudinal adherence in sliding windows [38] and CMA has been shown to produce adherence estimates comparable to other commonly used measures including MPR [46]. Adherence was only estimated for the drug metformin because unless contraindicated, metformin is the prescribed first line drug for the management of T2DM in South Africa [35]. In addition, diabetes treatment is done in an additive manner, where new drugs are added as a complement to the existing regimen (Fig 1), therefore estimating adherence using metformin only did not underestimate the measure in this study. The exclusion criteria of this study ensure that any patient who switches treatment due to metformin intolerance will not be considered in this analysis. In South Africa, routine treatment means that additional treatments may be added to metformin but are unlikely to be switched out with metformin.

Limited health services are an issue in South Africa as in other LMICs, where Practical Approach to Care Kit (PACK) guidelines are used and specialist referrals are only for patients with co-morbid disease or who need hospital admission. All people with T2DM are initiated on metformin by a doctor and thereafter are managed by clinician, nurses, and in some areas nurse- or community leader-led patient support groups. To monitor glycaemic control, the South African guidelines on the management of T2DM recommend HbA1c measures be done at the initial visit, then every 3–6 months annually thereafter [35]. However, in this study population, use of HbA1c to monitor glycaemic control was poorly implemented as most study participants had only one measure annually, while some were not having their HbA1c monitored at all (S2 Table). This situation is not unique to this study population as similar trends of low HbA1c implementation as a monitoring tool have also been observed elsewhere in South Africa [14, 47] and in Singapore [12]. In the current study, we observed median HbA1c values that were increasing over time even in those who were adherent to treatment (S2 Table). However, it might not be that HbA1c values in the population are increasing over time, but rather this observed trend could be because those who are accessing care and getting their HbA1c tested are those who are symptomatic and have chronic hyperglycaemia [14] that is more difficult to control. In addition, because the proportion of people with recorded HbA1c was decreasing every year (S2 Table), this could mean that the available HbA1c values do not accurately reflect what is happening in the population since they might be biased to those who have symptomatic hyperglycaemia. Since hyperglycaemia is largely asymptomatic, there is a need for regular monitoring of glycaemic control especially in ‘healthier’ asymptomatic individuals to prevent early onset of diabetes-related morbidity.

Polypharmacy and comorbidities have been reported as negative predictors of adherence [30]. In this study, however, we observed that individuals with chronic comorbidities that also required long term medication were likely to be adherent to their diabetes treatment. Similar findings were also observed in a study in Ethiopia where adherence to diabetes treatment increased with the number of non-diabetes medication an individual was prescribed [28]. This difference in observation maybe be because prior studies were done in older populations whereas our study population and the Ethiopian study population are generally younger with a median age of diabetes ascertainment less than 60 years (Table 1). The impact of age on adherence for T2D medication is unclear, and studies to date have had conflicting results [48]. Socioeconomic factors and population context may have a substantial effect on age dynamics, but additionally it can be difficult to assess the impact of younger age on chronic medication adherence as the majority of these diseases occur in later life. In addition, non-adherence due to polypharmacy is believed to be largely linked to medication costs, and in our study population the cost of treatment is not a barrier as health care is provided for free in public health facilities in South Africa. In addition, we also observed that people who were on a complex diabetes treatment regimen were more likely to be adherent (S2 Fig) than those on metformin only. This may be because these are people who might have more advanced disease and are therefore symptomatic and seeking and receiving more routine care. Results from a study using patient self-report in Ethiopia, however, found that participants who were on complex regimens were less likely to be adherent as they perceived themselves to be sicker and their situation helpless [24].

Results from the current study showed that individuals with long term adherence (trajectory D) had higher median health care encounters across all sliding windows in the two-year study observation period (S3 Table). Similar findings were seen in other studies where individuals who had frequent access to health care were more likely to be adherent to diabetes treatment [49]. In addition, in the current study PLWD on ART were more likely to have long term adherence. This might be because HIV infection in particular adherence to ART is managed through well-resourced programme in South Africa, therefore PLWHIV are accessing health care more [50] and are therefore likely to have better linkage to care for other comorbidities including diabetes. Given all these observations, it might be worth modelling the level of care given to PLWHIV in South Africa to PLWD. This could be particularly beneficial at the beginning of treatment since we observed that adherence in the first 4 months of initiating treatment was already less than 30% in this study population suggesting an urgent need for early intervention especially for those that initiate treatment while asymptomatic. Whilst no official posts for diabetes educators currently exist in Western Cape, in some facilities staff in nursing posts may be used to provide diabetes education. Diabetes councillors have been trained to provide lay education through patient support groups within the community. Patient education can provide an important route to assist with improving adherence trajectories and outcomes for PLWD in the Western Cape. The higher prevalence of T2DM in women in South Africa could be due to a higher prevalence of overweight or obesity in women as well as lower health-seeking behaviour in men [51, 52].

The use of routine data has made it possible to assess adherence in a very large virtual cohort of PLWD, and to understand some of the drivers of adherence across this large and diverse virtual population. Understanding real-world data in this way can provide insights into how healthcare clients access their medication and provide insights to design interventions to support healthcare clients in achieving better adherence trajectories.

## Supporting information

S1 Checklist The RECORD statement–checklist of items, extended from the STROBE statement, that should be reported in observational studies using routinely collected health data. (DOCX)

S1 Fig The five-criterion used to determine cluster partition choice. (TIF)

S2 Fig Impact of demographic factors, comorbidities, and dispensed diabetes drugs on participant metformin adherence trajectories. Odds Ratios (circles) with 95% Confidence Intervals (horizontal lines) are shown for each metformin adherence trajectory.
(TIF)

S1 Table Characteristics of the study population and stratified by HIV status. (DOCX)

S2 Table Median HbA1c values and proportion of study participants with HbA1c measures in the five years post diabetes ascertainment. (DOCX)

S3 Table Counts (%) and median (IQR) health facility encounters for study participants in the six months before starting diabetes treatment, in the two-year observation window (in four-month sliding windows) and in the 6 months after the two-year study observation window. (DOCX)
