# Supplementary figures and images for: Routine health data describe adherence and persistence patterns for oral diabetes medication for a virtual cohort in the Khayelitsha sub-district of Cape Town, South Africa
Source: PLOS Glob Public Health. 2023 Dec 21;3(12):e0002730. doi: 10.1371/journal.pgph.0002730 (PMC10734983; doi:10.1371/journal.pgph.0002730)

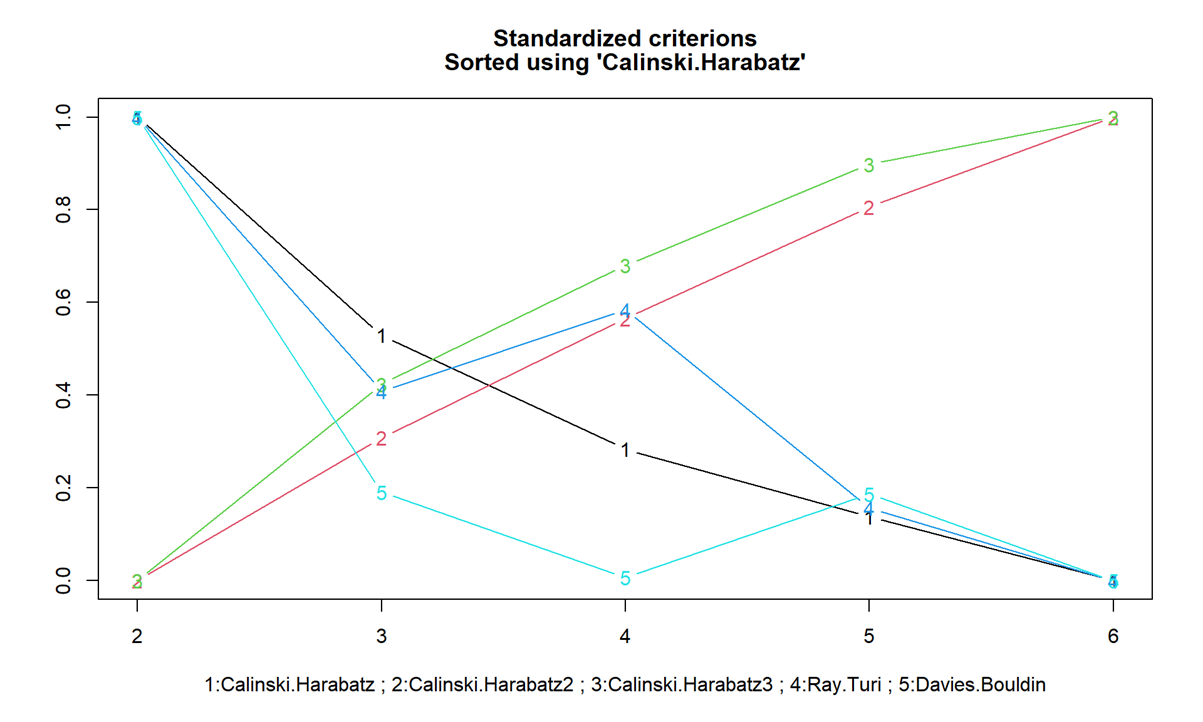

Supplement: S1 Fig — (TIF) [file pgph.0002730.s002.tif]

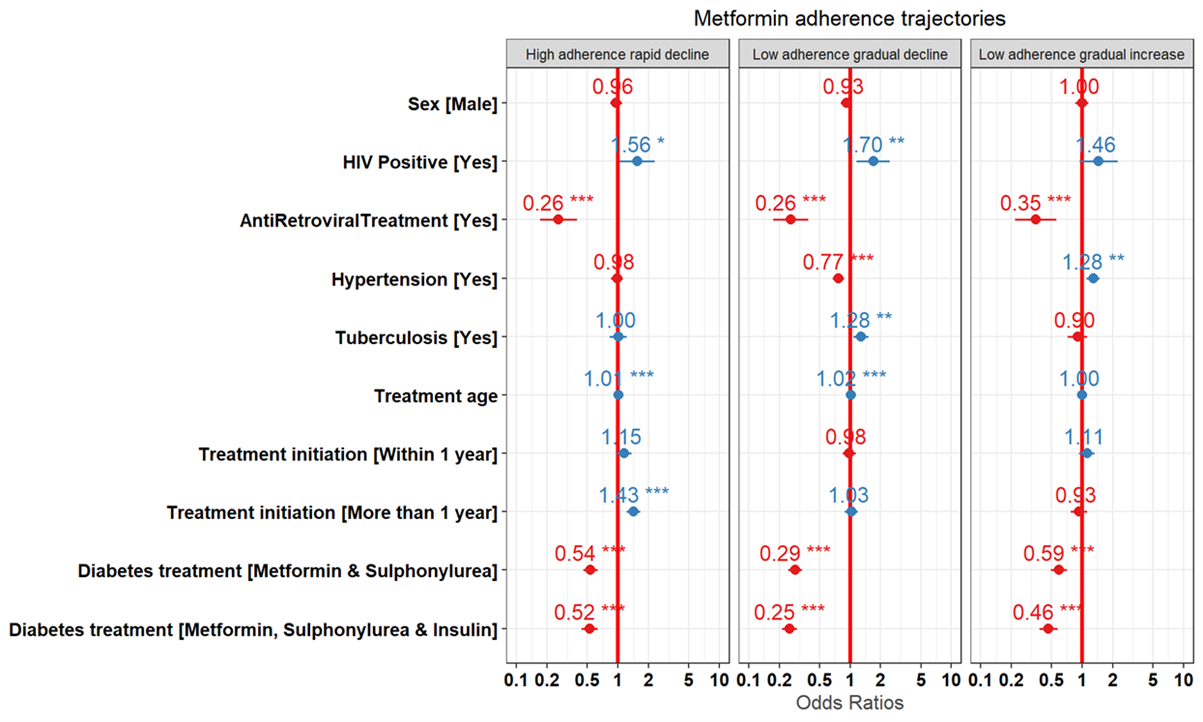

Supplement: S2 Fig — Odds Ratios (circles) with 95% Confidence Intervals (horizontal lines) are shown for each metformin adherence trajectory. (TIF) [file pgph.0002730.s003.tif]
