# Supplementary material for: Routine health data describe adherence and persistence patterns for oral diabetes medication for a virtual cohort in the Khayelitsha sub-district of Cape Town, South Africa
Source: PLOS Glob Public Health. 2023 Dec 21;3(12):e0002730. doi: 10.1371/journal.pgph.0002730 (PMC10734983; doi:10.1371/journal.pgph.0002730)
Supplement: S1 Table — (DOCX) [file pgph.0002730.s004.docx]

**S1 Table:** Characteristics of the study population and stratified by HIV status.

|  | **Whole study population**  *N=10541* | **HIV negative** *N=8969* | **HIV positive** *N=1572* | **P value** |
| --- | --- | --- | --- | --- |
| Sex: Female | 7053 (67.0%) | 5999 (67.0%) | 1054 (67.1%) | 0.947 |
| Diabetes Ascertainment Age (Years): | 52.0 [44.0;59.0] | 53.0 [45.0;61.0] | 46.0 [39.0;52.0] | <0.001 |
| Diabetes Treatment Initiation Age (Years): | 53.0 [45.0;60.0] | 54.0 [46.0;62.0] | 46.0 [40.0;53.0] | <0.001 |
| Diabetes Treatment Formulation: |  |  |  | 0.107 |
| Metformin only | 3525 (33.4%) | 2982 (33.2%) | 543 (34.5%) |  |
| Metformin & Sulphonylurea | 4417 (41.9%) | 3796 (42.3%) | 621 (39.5%) |  |
| Metformin, Sulphonylurea & Insulin | 2599 (24.7%) | 2191 (24.4%) | 408 (26.0%) |  |
| Diabetes Treatment Initiation: |  |  |  | <0.001 |
| At diabetes ascertainment | 5828 (55.3%) | 4936 (55.0%) | 892 (56.7%) |  |
| Within 1 year of ascertainment | 2156 (20.5%) | 1792 (20.0%) | 364 (23.2%) |  |
| More than 1 year after ascertainment | 2557 (24.3%) | 2241 (25.0%) | 316 (20.1%) |  |
| HIV Antiretroviral Treatment: | 1202 (11.4%) | 0 (0.0%) | 1202 (76.5%) | 0.000 |
| Cluster: |  |  |  | <0.001 |
| Adherent | 1544 (14.6%) | 1192 (13.3%) | 352 (22.4%) |  |
| Low adherence gradual decline | 4656 (44.2%) | 4005 (44.7%) | 651 (41.4%) |  |
| High adherence rapid decline | 2716 (25.8%) | 2380 (26.5%) | 336 (21.4%) |  |
| Low adherence gradual increase | 1625 (15.4%) | 1392 (15.5%) | 233 (14.8%) |  |
| Hypertension: | 6517 (61.8%) | 5713 (63.7%) | 804 (51.1%) | <0.001 |
| Tuberculosis: | 1385 (13.1%) | 850 (9.5%) | 535 (34.0%) | <0.001 |
