# Supplementary material for: Routine health data describe adherence and persistence patterns for oral diabetes medication for a virtual cohort in the Khayelitsha sub-district of Cape Town, South Africa
Source: PLOS Glob Public Health. 2023 Dec 21;3(12):e0002730. doi: 10.1371/journal.pgph.0002730 (PMC10734983; doi:10.1371/journal.pgph.0002730)
Supplement: S2 Table — (DOCX) [file pgph.0002730.s005.docx]

**S2 Table:** Median HbA1c values and proportion of study participants with HbA1c measures in the five years post diabetes ascertainment

|  | **Adherent (D)**  *N=1490* | **Low adherence gradual decline (A)**  *N=3740* | **High adherence rapid decline (B)**  *N=2591* | **Low adherence gradual increase (C)**  *N=1553* | **N** |  |
| --- | --- | --- | --- | --- | --- | --- |
| Year one  HbA1c (%) | 9.40 [7.60;11.8] | 9.00 [7.20;11.4] | 9.30 [7.40;11.7] | 9.10 [7.30;11.4] | 7676 |  |
| Participants with year one HbA1c | 1320 (88.6%) | 2881 (77.0%) | 2244 (86.6%) | 1231 (79.3%) | 9374 |  |
| Year two HbA1c | 8.80 [7.20;11.1] | 8.70 [7.10;11.1] | 8.70 [7.10;11.0] | 8.70 [7.20;11.0] | 4430 |  |
| Participants with year two HbA1c | 806 (54.1%) | 1431 (38.3%) | 1319 (50.9%) | 874 (56.3%) | 9374 |  |
| Year three HbA1c (%) | 9.10 [7.40;11.3] | 9.00 [7.30;11.4] | 9.20 [7.30;11.4] | 8.80 [7.30;11.1] | 4001 |  |
| Participants with year three HbA1c | 679 (45.6%) | 1451 (38.8%) | 1170 (45.2%) | 701 (45.1%) | 9374 |  |
| Year four HbA1c (%) | 9.50 [7.50;11.5] | 9.20 [7.30;11.4] | 9.60 [7.70;11.9] | 9.10 [7.40;11.3] | 3765 |  |
| Participants with year four HbA1c | 585 (39.3%) | 1420 (38.0%) | 1115 (43.0%) | 645 (41.5%) | 9374 |  |
| Year five HbA1c (%) | 9.80 [7.80;11.7] | 9.30 [7.50;11.3] | 9.70 [7.80;11.7] | 9.70 [7.60;11.4] | 3250 |  |
| Participants with year five HbA1c | 445 (29.9%) | 1304 (34.9%) | 985 (38.0%) | 516 (33.2%) | 9374 |  |

*N is the number of people in the study population who had an available HbA1c at the different time points.
