# Supplementary material for: Routine health data describe adherence and persistence patterns for oral diabetes medication for a virtual cohort in the Khayelitsha sub-district of Cape Town, South Africa
Source: PLOS Glob Public Health. 2023 Dec 21;3(12):e0002730. doi: 10.1371/journal.pgph.0002730 (PMC10734983; doi:10.1371/journal.pgph.0002730)
Supplement: S3 Table — (DOCX) [file pgph.0002730.s006.docx]

**S3 Table**: Counts (%) and median (IQR) health facility encounters for study participants in the six months before starting diabetes treatment, in the two-year observation window (in four-month sliding windows) and in the 6 months after the two-year study observation window.

|  | **Adherent (D)**  *N= 1544* | **Low adherence gradual decline (A)**  *N= 4655* | **High adherence rapid decline (B)**  *N= 2716* | **Low adherence gradual increase (C)**  *N= 1625* |
| --- | --- | --- | --- | --- |
| Six months before diabetes  treatment start (median, IQR) | 2.0 [1.0;4.0] | 2.0 [0.0;4.0] | 1.0 [0.0;3.0] | 2.0 [1.0;4.0] |
| Six months before diabetes  treatment start (%) | 1172 (75.9%) | 3309 (71.1%) | 1848 (68.0%) | 1248 (76.8% |
| Four months after diabetes  treatment start (median, IQR) | 5.0 [4.0;7.0] | 3.0 [1.0;5.0] | 4.0 [1.0;6.0] | 4.0 [2.0;6.0] |
| Four months after diabetes  treatment start (%) | 1497 (97.0%) | 4078 (87.6%) | 2239 (82.4%) | 1563 (96.2%) |
| Eight months after diabetes  treatment start (median, IQR) | 4.0 [2.0;5.0] | 1.0 [0.0;3.0] | 2.0 [1.0;4.0] | 2.0 [1.0;4.0] |
| Eight months after diabetes  treatment start (%) | 1488 (96.4%) | 2787 (59.9%) | 2270 (83.6%) | 1399 (86.1%) |
| Twelve months after diabetes  treatment start (median, IQR) | 4.0 [2.0;5.0] | 1.0 [0.0;3.0] | 2.0 [1.0;4.0] | 2.0 [1.0;4.0] |
| Twelve months after diabetes  treatment start (%) | 1449 (93.8%) | 2788 (59.9%) | 2291 (84.4%) | 1397 (86.0%) |
| Sixteen months after diabetes  treatment start (median, IQR) | 4.0 [2.0;5.0] | 1.0 [0.0;3.0] | 2.0 [1.0;4.0] | 3.0 [1.0;4.0] |
| Sixteen months after diabetes  treatment start (%) | 1382 (89.5%) | 2711 (58.2%) | 2218 (81.7%) | 1418 (87.3%) |
| Twenty months after diabetes  treatment start (median, IQR) | 3.0 [1.0;5.0] | 1.0 [0.0;2.0] | 2.0 [1.0;4.0] | 3.0 [1.0;5.0] |
| Twenty months after diabetes  treatment start (%) | 1251 (81.0%) | 2475 (53.2%) | 2058 (75.8%) | 1387 (85.4%) |
| Twenty-four months after diabetes  treatment start (median, IQR) | 3.0 [0.0;5.0] | 1.0 [0.0;3.0] | 2.0 [0.0;4.0] | 3.0 [1.0;4.0] |
| Twenty-four months after diabetes  treatment start (%) | 1141 (73.9%) | 2482 (53.3%) | 1964 (72.3%) | 1308 (80.5%) |
| Six months after study observation  window (median, IQR) | 4.0 [0.0;7.0] | 2.0 [0.0;5.0] | 4.0 [1.0;6.0] | 4.0 [1.0;6.0] |
| Six months after study observation  window (%) | 1093 (70.8%) | 2756 (59.2%) | 2038 (75.0%) | 1256 (77.3%) |
